# Role of Artificial Intelligence in Thyroid Cancer Diagnosis

**DOI:** 10.3390/jcm14072422

**Published:** 2025-04-02

**Authors:** Alessio Cece, Massimo Agresti, Nadia De Falco, Pasquale Sperlongano, Giancarlo Moccia, Pasquale Luongo, Francesco Miele, Alfredo Allaria, Francesco Torelli, Paola Bassi, Antonella Sciarra, Stefano Avenia, Paola Della Monica, Federica Colapietra, Marina Di Domenico, Ludovico Docimo, Domenico Parmeggiani

**Affiliations:** 1 Department of Integrated Activities in Surgery, Orthopedy and Hepato-Gastroenterology, Universitary Policlinico “Luigi Vanvitelli”, 80138 Naples, Italy; alessio.cece@unicampania.it (A.C.); massimo.agresti@unicampania.it (M.A.); nadia.defalco@unicampania.it (N.D.F.); pasquale.sperlongano@unicampania.it (P.S.); giancarlo.moccia@unicampania.it (G.M.); pasquale.luongo@unicampania.it (P.L.); francesco.miele@unicampania.it (F.M.); alfredo.allaria@unicampania.it (A.A.); francesco.torelli@unicampania.it (F.T.); paola.bassi@unicampania.it (P.B.); antonella.sciarra@unicampania.it (A.S.); 2 Department of Medicine and Surgery, University of Perugia, 06126 Perugia, Italy; stefano.avenia@unipg.it; 3 Department of Precision Medicine, University of Campania “Luigi Vanvitelli”, 80138 Naples, Italy; paola.dellamonica@unicampania.it (P.D.M.); federica.colapietra@unicampania.it (F.C.); marina.didomenico@unicampania.it (M.D.D.); 4 Department of General and Specialistic Surgery, Universitary Policlinico “Luigi Vanvitelli”, 80138 Naples, Italy; ludovico.docimo@unicampania.it

**Keywords:** thyroid cancer, artificial intelligence in thyroid nodule diagnosis, radiomics, machine learning, deep learning, genomic sequencing

## Abstract

The progress of artificial intelligence (AI), particularly its core algorithms—machine learning (ML) and deep learning (DL)—has been significant in the medical field, impacting both scientific research and clinical practice. These algorithms are now capable of analyzing ultrasound images, processing them, and providing outcomes, such as determining the benignity or malignancy of thyroid nodules. This integration into ultrasound machines is referred to as computer-aided diagnosis (CAD). The use of such software extends beyond ultrasound to include cytopathological and molecular assessments, enhancing the estimation of malignancy risk. AI’s considerable potential in cancer diagnosis and prevention is evident. This article provides an overview of AI models based on ML and DL algorithms used in thyroid diagnostics. Recent studies demonstrate their effectiveness and diagnostic role in ultrasound, pathology, and molecular fields. Notable advancements include content-based image retrieval (CBIR), enhanced saliency CBIR (SE-CBIR), Restore-Generative Adversarial Networks (GANs), and Vision Transformers (ViTs). These new algorithms show remarkable results, indicating their potential as diagnostic and prognostic tools for thyroid pathology. The future trend points to these AI systems becoming the preferred choice for thyroid diagnostics.

## 1. Introduction

The growing evolution of artificial intelligence (AI) algorithms has been accompanied by their increasing use in the various stages of thyroid nodule diagnosis, ranging from ultrasound to cytopathological and molecular evaluation. Thyroid cancer, the most common endocrine tumor, is more prevalent in females, with a male-to-female ratio of 3:1. In recent decades, the incidence of thyroid cancer has progressively increased, with a substantially stable mortality rate due to over-diagnosis.

Global incidence estimates report approximately 450,000 new cases of thyroid cancer in women and 150,000 in men (GLOBOCAN 2020 data) [1,2]. The Italian epidemiological situation mirrors this global trend, with thyroid neoplasms ranked fourth among the most frequent cancers in women, and second in the 0–49 age group. In men, thyroid cancer is the fifth most frequent neoplasm in Italy [3,4]. Despite the high incidence, the annual mortality rate remains stably low, at 0.5/100,000 [1]. Given the significant incidence, it is essential to achieve early diagnosis through appropriate diagnostic procedures for effective therapeutic management. Additionally, providing an early prognostic judgment to define the patient’s risk profile at the pre-operative stage is crucial to guide subsequent therapeutic choices, such as surgery or vigilant observation.

The scientific community agrees on the need for a personalized diagnostic and therapeutic pathway tailored to each individual patient. This review will highlight the role of artificial intelligence systems as a powerful diagnostic and prognostic tool to ensure personalized treatment. In thyroid diagnostics, traditional machine learning (ML) and deep learning (DL) algorithms remain valid for providing diagnostic and prognostic support. However, new models have proven to be equally, if not more, effective compared to traditional ones in various applications; however, early diagnosis and accurate prognostic assessment remain challenging, particularly in distinguishing aggressive from indolent tumors. In this context, AI has the potential to enhance risk stratification and optimize therapeutic decisions, addressing these critical issues and suggesting a potential future extension of their use in the prevention of thyroid cancer.

## 2. Materials and Methods

This literature review was conducted by searching for articles published in PUBMED from 2018 to 2024. A total of 33 articles met the inclusion criteria and were analyzed. The articles included systematic reviews, narrative reviews, meta-analyses, prospective studies, retrospective studies, cohort studies, comparative diagnostic studies, experimental cohort studies with validation, retrospective observational studies, validation studies, and blind, multicenter prospective studies. The epidemiology of thyroid cancer was referenced from the AIOM guidelines (2017 edition, updated in October 2019) and the GLOBOCAN 2020 data from the European Society of Endocrinology.

## 3. Artificial Intelligence in Thyroid Nodule Diagnostics

### AI Applied to Ultrasound Imaging

The ultrasound features considered indicators of risk, when analyzing a thyroid nodule, are hypo-/hyper-echogenicity, shape, margins, microcalcifications, structural pattern, and composition. These characteristics, in particular echogenicity, echo-structure, and composition, in classical ultrasound practice, are inferred through processes including texture analysis and quantitative echo-structure assessment [5]. This first requires that a region of interest (ROI) is selected within the ultrasound image by means of segmentation, which is followed by image processing and finally by obtaining data on texture, shape, and surrounding ratios [5,6]. In this context, various machine learning and deep learning algorithms have found applications and are nowadays integrated and used. Several works certify the validity of artificial intelligence systems; in particular, Liang et al. [7] demonstrated the validity of a risk score for thyroid lesions based on radiomic analysis. The model, in this case a machine learning-based system, demonstrated sensitivity and specificity comparable to those obtained by applying ACR-TIRADS criteria, in the classification of thyroid lesions [7]. The study by Chunquan et al. [8], on the other hand, evaluated the potential of deep learning-based systems. In particular, a system capable of detecting differentiated thyroid carcinoma was set up, based on both image and video elements. Such a model for analyzing video recordings showed superior accuracy in detecting malignant thyroid lesions compared to experienced radiologists, although one of the limitations of this system, as of all those based on deep learning technologies, is represented by the fact that the processes through which the algorithm arrives at a conclusion are substantially unknown. The study attempted to overcome this limitation by highlighting, through a color code, the areas of the image that were most taken into account by the algorithm. This made it possible not only to assess the activity of the system in expressing a prognostic judgment but also to identify those areas, hence the characteristics, of the lesion that were most taken into account for diagnostic evaluation [8]. Similarly, we highlight the good performance of the algorithm developed by the Duke University School of Medicine [9], a software based on deep learning technology capable of establishing a malignancy percentage for each thyroid lesion examined, subsequently classifying it according to risk levels and indicating the need, or at least, the recommendation to perform a biopsy. The algorithm showed higher sensitivity than that achieved by radiologists by applying ACR-TIRADS criteria [10].

## 4. AI in Thyroid Nodule Classification

The role of artificial intelligence, machine learning, and deep learning systems integrated into computer-aided diagnosis tools is to assist clinicians in the diagnostic process, enhancing lesion recognition and evaluation while sometimes revealing unexpected features. These systems provide additional diagnostic insights, improve accuracy, and reduce image interpretation time [6].

These CAD (Table 1) (computer-aided diagnosis) systems demonstrate sensitivity and specificity levels of approximately 88% and 81%, respectively, for distinguishing between benign and malignant thyroid lesions [11]. Their effectiveness has been evaluated in multiple studies. For instance, Li Y. et al. [12] compared the diagnostic performance of the S-Detect 2 system (Samsung Medison Co., Ltd., Seoul, Republic of Korea) with that of twelve practitioners, including three experienced radiologists and nine physicians in specialist training. The results showed that the system’s sensitivity and specificity were comparable to those of experienced radiologists and superior to those of less-experienced physicians. Notably, while CAD assistance did not significantly impact expert radiologists’ diagnoses, it improved the performance of junior doctors by increasing their sensitivity [12]. Another relevant study by Fresilli D. et al. [13] compared the S-Detect system with a radiologist with over ten years of experience, a physician in specialist training, and a medical student with basic ultrasound knowledge. The findings confirmed the system’s utility, particularly for less-experienced physicians, despite some limitations, such as the significant impact of segmentation accuracy on diagnostic outcomes.

Another CAD-tested system is AIBx software (version 1), which retrieves images from a predefined dataset to identify lesions similar to the one being analyzed and classify them as benign or malignant. AIBx demonstrated greater prognostic accuracy than the EU-TIRADS criteria [14].

## 5. Limitations

Notwithstanding the valid results obtained by these technologies, certain limitations of these methods must, however, be taken into account. First and foremost, there are ethical–legal concerns related to diagnoses based on algorithms, particularly in deep learning systems, where the decision-making processes remain largely opaque. Key considerations include patient consent, transparency in artificial intelligence decision making, data privacy, and accountability for diagnostic errors. Ensuring that artificial intelligence-driven diagnoses align with ethical and legal standards remains a critical challenge in clinical adoption. Another noteworthy limitation, specifically in the ultrasound field, is the variability among different ultrasound probes and machines. Differences in hardware can result in significant alterations in image quality, which may affect the artificial intelligence system’s ability to accurately analyze images. While some artificial intelligence models can be trained to account for such variability, additional standardization measures or calibration techniques may be necessary to maintain consistency across different imaging devices. A recurring limitation, present in all reported studies, is the study population. A clear example of this is the study by Li et al. [12], in which the percentage of nodules that turned out to be malignant on histological examination was significantly higher in lesions ≤ 1.5 cm than in larger ones. This is a consequence of the fact that the study was conducted exclusively on a surgical patient population, i.e., individuals who underwent thyroidectomy and had histological verification. The question remains whether machine learning and deep learning algorithms trained on this specific population maintain the same accuracy when applied to broader and more diverse patient groups [12]. To address this, artificial intelligence models should undergo rigorous validation across different demographic and clinical cohorts to ensure their generalizability and reliability in varied real-world settings [15].

## 6. AI Applications in Pathological Thyroid Diagnosis

Machine learning models have been explored to improve the cytopathological analysis of thyroid aspirate (FNAC). For example, Guan et al. [16] employed a deep convolutional neural network (DCNN) called VGG-16 to perform differential diagnosis between papillary thyroid carcinoma (PTC) and benign nodules using digital images of FNAC slides stained with H&E. Overall, 279 images from 179 cases (159 PTC and 120 benign) were analyzed and fragmented, bringing the total to 887 images. Histological diagnosis was used for PTC cases, while cytology was used for benign nodules. DCNN showed an accuracy of 97.66% on fragmented images, an accuracy of 95% at the patient level, a sensitivity of 100%, and a specificity of 94.91%, which were higher than the conventional FNAC (accuracy of 89–95%) [17]. In another study, Sanyal et al. [18] used a convolutional neural network (CNN) model to differentiate between PTC and non-PTC nodules. They used 370 images (186 PTC and 184 non-PTC), taken from 20 cytological slides of 20 patients, to train the software. Using the cytological diagnosis as a reference, the CNN model showed a diagnostic accuracy of 85%. The main limitation found was the number of false positives due to errors in identifying normal follicular cells and areas of dense colloid as PTC. To overcome the need for manual segmentation, Range et al. [19] developed an automated classification system using whole slide images to predict malignancy. In the first step, a CNN selected the area of interest containing follicular cells. Subsequently, the CNN applied to this region predicted the final pathology. A total of 799 slides were used to train the model, which was then tested on 109 slides, resulting in an AUC of 0.931, comparable to that of an experienced cytopathologist (0.932), with a specificity of 90.5% and a sensitivity of 92.0%. Another innovative approach was taken by Maleki S et al. [20] who used a support vector model (SVM) to analyze microscopic descriptions in cytopathology reports, distinguishing between non-invasive follicular neoplasms and neoplasms with PTC-like features (NIFTP) and classical PTC (cPTC). Descriptions, with 59 cytological keywords grouped into 32 features, were analyzed, resulting in an AUC of 0.76, with a sensitivity of 72.6% and a specificity of 81.6%. The model accurately differentiated NIFTP from cPTC in about 75% of the cases. The use of artificial intelligence to predict mutations, tumor composition, and prognosis from histopathological images is a promising field. Fu et al. [21] were able to predict the presence of a BRAF-positive mutation in histopathological slides of the thyroid. A recent review on the use of AI in thyroid cytopathology [22] concluded that the studies reviewed showed comparable performance to pathologists, with faster and more easily performed artificial intelligence methods. However, most institutions do not digitize every slide due to a lack of equipment and the cost of archiving images, thus limiting the application of AI in cytopathology until this practice becomes commonplace.

## 7. Applications of Artificial Intelligence in the Molecular Evaluation of Thyroid Nodules

Approximately 20% of thyroid nodules biopsied may be indeterminate [23]. To address this challenge, molecular marker tests have been developed to improve risk stratification for these nodules, providing more accurate predictions regarding malignancy. One of the earliest and most notable advancements in this area is the Afirma^®^ Genomic Sequencing Classifier (GSC), which integrates machine learning into clinical thyroidology applications. This test employs a series of machine learning classifiers to analyze data derived from gene expression patterns associated with both benign and malignant thyroid nodules [24,25]. The molecular mechanisms behind this test rely on the identification of specific genetic signatures that differentiate between benign and malignant tissue. However, the initial version of the test, while sensitive, lacked specificity, particularly in identifying Hürthle cell tumors. In response to this limitation, the latest iteration of the Afirma^®^ test incorporated two critical indices: the Hürthle cell index and the Hürthle cell neoplasm index. These indices, based on Support Vector Machine (SVM) algorithms, were developed to enhance specificity in predicting Hürthle cell tumors [26]. By integrating these additional features, the test significantly improved its diagnostic accuracy, offering better clinical decision-making tools for endocrinologists.

Another significant advancement in molecular testing for thyroid nodules is the ThyroSeq test. The current version (version 3) employs next-generation sequencing to analyze 112 genes associated with thyroid cancer, utilizing a decision tree-based algorithm to estimate the likelihood of malignancy in a given nodule. The test’s decision-making process is grounded in the identification of genetic alterations known to be associated with various thyroid malignancies. This algorithm has been extensively validated in prospective, multi-center, double-blind studies, demonstrating its reliability and applicability in clinical practice [27,28].

These advancements in molecular testing not only improve the accuracy of thyroid nodule diagnosis but also have significant clinical implications. They offer patients a more personalized approach to diagnosis, potentially reducing the need for invasive surgical procedures in cases of benign nodules. Furthermore, these tests could pave the way for more targeted therapeutic strategies, as precise molecular profiling allows clinicians to better predict the aggressiveness of a tumor and tailor treatment accordingly.

## 8. Content-Based Image Retrieval (CBIR) and with Enhanced Saliency (SE-CBIR)

The CBIR (content-based image retrieval) model and its evolution into SE-CBIR (CBIR with enhanced saliency) are clear examples of advanced artificial intelligence systems that have been tested and have produced encouraging results in clinical diagnostics, especially in the pathological context. CBIR is defined as the procedure, based on a classical convolutional neural network (CNN) system, of searching images from histopathological databases. This system, in order to reach a histopathological diagnosis, analyzes and retrieves, in a short time, cases similar to the one under investigation, from a database of known images. The degree of similarity between the images under examination and those available in the database is quantified thanks to the information retrieved by a deep neural network, part of the system, which precisely describes all features of the histopathological image. The SE-CBIR system represents a further development, consistent with the most recent published literature on the subject [29]. It is a new image retrieval model, based on saliency maps, i.e., computer vision methods that highlight the most relevant parts of the image, based on the prediction made by the neural network. The result is a more accurate image retrieval and an overall optimized procedure. The validity of the SE-CBIR system was demonstrated in a study conducted at the Dermatology Clinic of the University Hospital of Zurich [29]. The image retrieval accuracy achieved by the SE-CBIR system was 84, i.e., 7% higher than that achieved by the conventional CBIR system (77%). In addition, it was possible to reduce the number of non-melanoma lesions previously diagnosed as melanoma by 53% through the use of SE-CBIR as a supporting tool. Thus, as stated by the study under review, SE-CBIR has a better recovery accuracy than traditional CBIR approaches based on the CNN [29].

## 9. Restore-Generative Adversarial Network

In the field of clinical diagnosis and pathological anatomy, deep learning algorithms have achieved significant results in imaging analysis, such as the segmentation of neoplastic areas, the detection of nuclei, and more. However, in clinical practice, these algorithms often face challenges, particularly when it comes to image quality issues such as low resolution, blurring, and color variations. In this context, the Restore-GAN (Restore-Generative Adversarial Network) model offers a solution by overcoming these limitations, enhancing image quality, improving resolution, reducing blurring, and addressing color variations. The results from studies using the Restore-GAN are encouraging, demonstrating its potential to significantly improve image clarity and diagnostic accuracy.

Notably, the Restore-GAN model’s performance has been validated in both experimental and clinical settings, showcasing its robustness in real-world applications. The study conducted by Rong et al. (2023) [30], for example, tested the model on a large set of pathological images, further supporting its practical utility. This study utilized a comprehensive dataset, highlighting the model’s ability to function effectively in a diverse range of clinical scenarios, thus presenting it as a valuable tool for advancing digital pathology. The findings of this study, observed in a clinical context, underline the potential of the Restore-GAN model to facilitate the integration of deep learning in routine diagnostic workflows, ultimately enhancing the efficiency and accuracy of pathological assessments.

## 10. Vision Transformers and Application in Thyroid Diagnostics

A Vision Transformer (ViT) is a type of neural network that differs significantly from classical convolutional neural networks (CNNs). Rather than processing an image as a set of pixels, the ViT breaks the image down into smaller patches and processes these patches sequentially. This sequence of patches is flattened and then embedded by the transformer’s encoder, which maintains position embeddings, i.e., spatial information. The model captures the relationships between these patches, ensuring that contextual dependencies across the entire image are recognized. This approach contrasts with traditional CNNs, which focus on local pixel-based features, offering ViTs a unique advantage in processing more complex patterns in data.

In medical imaging, ViT-based models have recently outperformed CNN-based systems in several applications. ViTs have shown a greater ability to capture long-range dependencies and store more information, albeit with the trade-off of requiring a larger amount of data for training [31]. One notable application where ViT systems have demonstrated superiority is in breast ultrasound imaging. The study by Ayana G and Choe SW, titled “BUViTNet: Breast Ultrasound Detection via Vision Transformers” [31], highlighted the effectiveness of a ViT-based system in detecting breast abnormalities. The algorithm was trained using breast ultrasound image datasets, and its classification performance was rigorously evaluated. The results revealed that ViT-based systems significantly outperformed traditional CNN-based models, demonstrating their potential to act as a powerful diagnostic aid when used in appropriate clinical settings [31].

**Figure 1:**
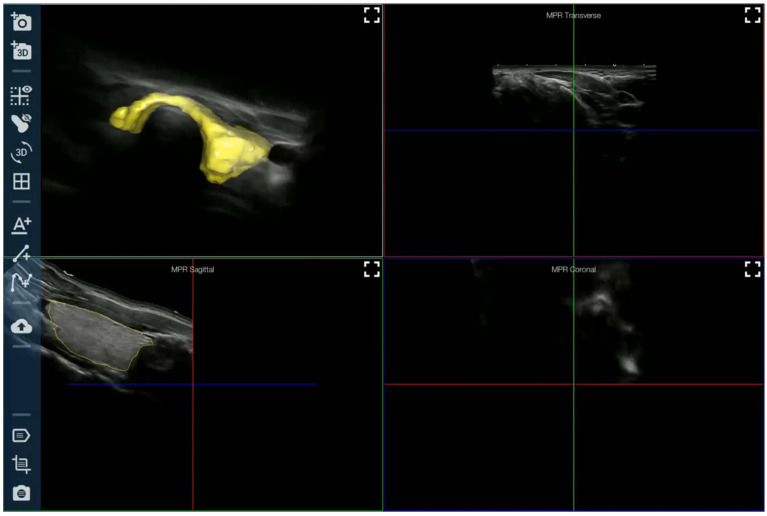
Three-dimensional representation of an ultrasound image of a thyroid nodule [32].

In thyroid diagnostics, machine learning (ML) and deep learning (DL) algorithms remain the predominant tools for diagnostic assistance. However, ViT-based systems have shown promising results in this field, suggesting their potential for broader implementation. A significant example is the ViT-based thyroid nodule classification system developed by Sun J et al. [32]. This system addresses a key challenge in thyroid imaging: the difficulty in differentiating between benign and malignant nodules, particularly those classified as TI-RADS level 3. This has historically led to inconsistent diagnostic interpretations, an overuse of biopsies, and, at times, overdiagnosis. The model proposed by Sun J et al. [32], called the CT-ViT, leverages contrastive learning techniques to optimize both global and local characterization of lesions. In doing so, it enables accurate differentiation between malignant nodules and those classified as TI-RADS 3, improving the overall diagnostic accuracy. The ViT-based system outperforms traditional deep learning networks in classifying thyroid nodules from radiological images, including ultrasound. This capability positions ViTs as a valuable tool in computer-aided diagnosis, with the potential for increasing integration into clinical workflows for more accurate thyroid diagnostics [32] (Figure 1).

## 11. Conclusions

- Advancement in diagnosis with AI: The integration of artificial intelligence (AI), particularly through machine learning (ML) and deep learning (DL) algorithms, represents a significant step forward in the diagnosis and prognosis of thyroid nodules.
- Comparable and superior performance: The studies analyzed in this review show that AI-based systems have achieved levels of accuracy, sensitivity, and specificity comparable to, and in some cases superior to, those achieved by experienced practitioners in the fields of ultrasound, cytopathological, and molecular diagnostics.
- Improvement in diagnostic efficiency: artificial intelligence algorithms have been successfully tested in the evaluation of ultrasound images of thyroid nodules, the classification of lesions, and the prediction of malignancy, showing the ability to improve diagnostic efficiency and reduce evaluation time.
- Clinically approved systems: Systems such as Am-CAD-UT, Koios DS Thyroid, MEDOThyroid, and S-Detect have obtained significant approvals for clinical use, highlighting the reliability and usefulness of these tools in supporting clinicians, especially less experienced ones.
- Improvements in cytopathological and molecular diagnostics: In the field of cytopathological and molecular diagnostics, the use of artificial intelligence has improved accuracy in differentiating between benign and malignant lesions and in predicting genetic mutations, contributing to more precise risk stratification and personalization of therapies.
- Future evolution of AI systems: Looking forward, the continued evolution of artificial intelligence systems, including new models such as enhanced saliency CBIR (SE-CBIR), Restore-Generative Adversarial Network (GAN) models, and Vision Transformers (ViTs), promises to further expand the applications of AI in thyroid diagnostics.
- Benefits for patients and healthcare systems: These advancements not only improve the quality of diagnosis but also open the way to new possibilities in the prevention and personalized management of thyroid cancer, with potentially significant benefits for patients and the healthcare system.

## Figures and Tables

**Table 1:** Thyroid nodule diagnostic support systems that have requested approval from the Food and Drug Administration [10].

CAD SYSTEM	SOURCE DATA	NODULE RECOGNITION	TYPE OF APPLICATION
AmCAD-UT (AmCad BioMed Corporation, Taipei City, Taiwan);	Single image	Semi-automatic	Local computer
Koios DS Thyroid (Koios Medical, Inc., New York, NY, USA);	Double projection image	Manual	Local server
MEDO-Thyroid (MEDO AI, Edmonton, AB, Canada);	Video	Manual	Cloud
S-Detect (Samsung Medison Co., Ltd., Seoul, Republic of Korea)	Single image	Manual	Installed in the ultrasound machine

## References

[1] Pizzato M., Li M., Vignat J., Laversanne M., Singh D., La Vecchia C., Vaccarella S. (2022). The epidemiological landscape of thyroid cancer worldwide: GLOBOCAN estimates for incidence and mortality rates in 2020. Lancet Diabetes Endocrinol..

[2] (2022). AME NEWS No. 66-August 2022.

[3] Locati L.D., Bernardo G. (2022). Thyroid. Cancer Numbers in Italy 2022.

[4] Barp S., Grani G. (2017). Thyroid Cancer Guidelines.

[5] Sollini M., Cozzi L., Chiti A., Kirienko M. (2018). Texture analysis and machine learning to characterise suspected thyroid nodules and differentiated thyroid cancer: Where do we stand?. Eur. J. Radiol..

[6] Bini F., Pica A., Azzimonti L., Giusti A., Ruinelli L., Marinozzi F., Trimboli P. (2021). Artificial intelligence in the thyroid field—A comprehensive review. Cancers.

[7] Liang J., Huang X., Hu H., Liu Y., Zhou Q., Cao Q., Wang W., Liu B., Zheng Y., Li X. (2018). Predicting malignancy in thyroid nodules: Radiomics score versus 2017 American College of Radiology Thyroid Imaging, Reporting and Data System. Thyroid.

[8] Zhang C., Liu D., Huang L., Zhao Y., Chen L., Guo Y. (2022). Classification of thyroid nodules by using deep learning radiomics based on ultrasound dynamic video. J. Ultrasound Med..

[9] Yang J., Page L.C., Wagner L., Wildman-Tobriner B., Bisset L., Frush D., Mazurowski M.A. (2023). Thyroid nodules on ultrasound in children and young adults: Comparison of diagnostic performance of radiologists’ impressions, ACR TI-RADS, and a deep learning algorithm. Am. J. Roentgenol..

[10] Barp S., Grani G. (2023). Intelligenza artificiale nella diagnostica del nodulo tiroideo. L’Endocrinologo.

[11] Xue Y., Zhou Y., Wang T., Chen H., Wu L., Ling H., Wang H., Qiu L., Ye D., Wang B. (2022). Accuracy of ultrasound diagnosis of thyroid nodules based on artificial intelligence-assisted diagnostic technology: A systematic review and meta-analysis. Int. J. Endocrinol..

[12] Li Y., Liu Y., Xiao J., Yan L., Yang Z., Li X., Zhang M., Luo Y. (2023). Clinical value of artificial intelligence in thyroid ultrasound: A prospective study from the real world. Eur. Radiol..

[13] Fresilli D., Grani G., De Pascali M.L., Alagna G., Tassone E., Ramundo V., Ascoli V., Bosco D., Biffoni M., Bononi M. (2020). Computer- aided diagnostic system for thyroid nodule sonographic evaluation outperforms the specificity of less experienced examiners. J. Ultrasound.

[14] Swan K.Z., Thomas J., Nielsen V.E., Jespersen M.L., Bonnema S.J. (2022). External validation of AIBx, an artificial intelligence model for risk stratification, in thyroid nodules. Eur. Thyroid J..

[15] Thomas J., Ledger G.A., Mamillapalli C.K. (2020). Use of artificial intelligence and machine learning for estimating malignancy risk of thyroid nodules. Curr. Opin. Endocrinol. Diabetes Obes..

[16] Guan Q., Wang Y., Ping B., Li D., Du J., Qin Y., Lu H., Wan X., Xiang J. (2019). Deep convolutional neural network VGG16 model for differential diagnosing of papillary thyroid carcinomas in cytological images: A pilot study. J. Cancer.

[17] Bongiovanni M., Spitale A., Faquin W.C., Mazzucchelli L., Baloch Z.W. (2012). The Bethesda System for Reporting Thyroid Cytopathology: A meta-analysis. Acta Cytol..

[18] Sanyal P., Mukherjee T., Barui S., Das A., Gangopadhyay P. (2018). Artificial intelligence in cytopathology: A neural network to identify papillary carcinoma on thyroid fine-needle aspiration cytology smears. J. Pathol. Inform..

[19] Elliott Range D.D., Dov D., Kovalsky S.Z., Henao R., Carin L., Cohen J. (2020). Application of a machine learning algorithm to predict malignancy in thyroid cytopathology. Cancer Cytopathol..

[20] Maleki S., Zandvakili A., Gera S., Khutti S.D., Gersten A., Khader S.N. (2019). Differentiating noninvasive follicular thyroid neoplasm with papillary-like nuclear features from classic papillary thyroid carcinoma: Analysis of cytomorphologic descriptions using a novel machinelearning approach. J. Pathol. Inform..

[21] Fu Y., Jung A.W., Torne R.V., Gonzalez S., Vöhringer H., Shmatko A., Yates L.R., Jimenez-Linan M., Moore L., Gerstung M. (2020). Pan-cancer computational histopathology reveals mutations, tumor composition and prognosis. Nat. Cancer.

[22] Girolami I., Marletta S., Pantanowitz L., Torresani E., Ghimenton C., Barbareschis M., Scarpa A., Brunelli M., Barresi V., Trimboli P. (2020). Impact of image analysis and artificial intelligence in thyroid pathology, with particular reference to cytological aspects. Cytopathology.

[23] Bongiovanni M., Bellevicine C., Troncone G., Sykiotis G.P. (2019). Approach to cytological indeterminate thyroid nodules. Gland. Surg..

[24] Diggans J., Kim S.Y., Hu Z., Pankratz D., Wong M., Reynolds J., Tom E., Pagan M., Monroe R., Rosai J. (2015). Machine learning from concept to clinic: Reliable detection of BRAF V600E DNA mutations in thyroid nodules using highdimensional RNA expression data. Pac. Symp. Biocomput..

[25] Hao Y., Choi Y., Babiarz J.E., Kloos R.T., Kennedy G.C., Huang J., Walsh P.S. (2019). Analytical Verification Performance of A firma Genomic Sequencing Classifier in the Diagnosis of Cytologically Indeterminate Thyroid Nodules. Front. Endocrinol..

[26] Hao Y., Duh Q.-Y., Kloos R.T., Babiarz J., Harrell R.M., Traweek S.T., Kim S.Y., Fedorowicz G., Walsh P.S., Sadow P.M. (2019). Identification of Hürthle cell cancers: Solving a clinical challenge with genomic sequencing and a trio of machine learning algorithms. BMC Syst. Biol..

[27] Steward D.L., Carty S.E., Sippel R.S., Yang S.P., Sosa J.A., Sipos J.A., Figge J.J., Mandel S., Haugen B.R., Burman K.D. (2019). Performance of a Multigene Genomic Classifier in Thyroid Nodules with Indeterminate Cytology: A Prospective Blinded Multicenter Study. JAMA Oncol..

[28] Mallick U.K., Mazzaferri E.L., Harmer C., Kendall-Taylor P. (2018). Practical management of thyroid cancer: A multidisciplinary approach. Practical Management of Thyroid Cancer: A Multidisciplinary Approach.

[29] Gassner M., Barranco Garcia J., Tanadini-Lang S., Bertoldo F., Fröhlich F., Guckenberger M., Haueis S., Pelzer C., Reyes M., Schmithausen P. (2023). Saliency-Enhanced ContentBased Image Retrieval for Diagnosis Support in Dermatology Consultation: Reader Study. JMIR Dermatol..

[30] Rong R., Wang S., Zhang X., Wen Z., Cheng X., Jia L., Yang D.M., Xie Y., Zhan X., Xiao G. (2023). Enhanced Pathology Image Quality with Restore-Generative Adversarial Network. Am. J. Pathol..

[31] Ayana G., Choe S.W. (2022). BUViTNet: Breast Ultrasound Detection via Vision Transformers. Diagnostics.

[32] Sun J., Wu B., Zhao T., Gao L., Xie K., Lin T., Sui J., Li X., Wu X., Ni X. (2022). Classification for thyroid nodule using ViT with contrastive learning in ultrasound images. Comput. Biol. Med..

